# Supplementary material for: Chromothripsis during telomere crisis is independent of NHEJ, and consistent with a replicative origin
Source: Genome Res. 2019 May;29(5):737–49. doi: 10.1101/gr.240705.118 (PMC6499312; doi:10.1101/gr.240705.118)
Supplement: Supplemental Material [file supp_gr.240705.118_Supplemental_file_1.zip › contigs/annotated_contigs/DB85/contig.2.DB85_length_657_mean_cov_7.98477929985.docx]

**DB85_length_657_mean_cov_7.98477929985**

```
TATCTGCTACAGACAATAAAAATTATTATCTCTTAAAATAACTCCAAACAGAAAAATAACTAGACATAGTGGCTGCACTGTACATTGTG
 >chr3:187299301-187299678 + E=5e-194
TTCATTCATGAAAACAATTATGGCGCTAAATAAAAAGACAATAGAAATTATTTTCTTCTTTTTGTTTAAACTTTTATTTTAGGTTCAGG

GGTACATGTACAGGTTTATTATATAGGTATTCATGTCATGGGGTTTGTTGTGCAGATTATTTCATCACTCAGATACTAAGTCTAGTACC

CAATAGCTATTTTTTCTGATTCTCTCCCTCCTCCAACCCTCCACCCACCAGTATCCCCCAGTGTCTGTTGCTCCCCTCTTTGTGTCCAT

ATGTTCTCATCATTTATCATC|TGACTGAGCTGCTGAACACACATCGCTGTGCTATTTCATCCTACTCAGCTTAGCACTTATGACCCCC
                       >chr3:187480262-187480542 + E=1e-156
TGTGACTATTTAGTAATTGCACTGTCTCTCCTCTTGAATGTGAGTGCCTGGAGGTCAGTAGCTATTTGTTTCTTCTCCTTGGTATCTTA

ATCCTCTCCTGGTGCCTAAGATAAGCAACTTACCCATCTCGCACATGTTTTGAAGGAAGAGGACCCAATAGACTGAGACTTTTACTGGA

GTTTATGCACCTTTATTTATCTTCTCATATTTTCA
```
